# Investigating the Influence of Interaction on Learning Persistence in Online Settings: Moderation or Mediation of Academic Emotions?

**DOI:** 10.3390/ijerph17072320

**Published:** 2020-03-30

**Authors:** Jianhui Yu, Changqin Huang, Zhongmei Han, Tao He, Ming Li

**Affiliations:** 1 Department of Educational Technology, Zhejiang Normal University, Jinhua 321004, China; jianhuiyu@126.com (J.Y.); zmhan@m.scnu.edu.cn (Z.H.); mingli@zjnu.edu.cn (M.L.); 2 School of Information Technology in Education, South China Normal University, Guangzhou 510631, China; tao.he2016@gmail.com

**Keywords:** learning persistence, students’ interaction, academic emotions, mediating effect, moderating effect

## Abstract

Learning persistence is a critical element for successful online learning. The evidence provided by psychologists and educators has shown that students’ interaction (student-student (SS) interaction, student-instructor (SI) interaction, and student-content (SC) interaction) significantly affects their learning persistence, which is also related to their academic emotions. However, few studies explore the relations among students’ interaction, academic emotions and learning persistence in online learning environments. Furthermore, no research has focused on multi-dimensional students’ interaction and specific academic emotions. Based on person-environment interaction model and transactional distance theory, this study investigates the relationship between students’ interaction and learning persistence from the perspective of moderation and mediation of academic emotions including enjoyment, boredom, and anxiety. Data were collected from 339 students who had online learning experience in China. AMOS 22.0 (IBM, Armonk, NY, USA) and SPSS 22.0 (IBM, Armonk, NY, USA) were employed to analyze the mediating and moderating effects of academic emotions, respectively. The results revealed that students’ interaction and academic emotions directly related to learning persistence. Specifically, enjoyment, anxiety and boredom had significant mediating and moderating effects on the relationship between students’ interaction and learning persistence. Based on these findings, we further discussed the theoretical and practical implications on how to facilitate students’ learning persistence in online learning environments.

## 1. Introduction

As a common way of learning, online learning has attracted lots of scholarly interest. Based on the well-established fact that online learning suffers from a low level of learning persistence, the question then arises as to how learning engagement and persistence are facilitated in online learning environments. This is a critical problem that remains unsolved [1]. Previous research has indicated that learning persistence had a great positive influence on boosting learning performance and achievement [2,3]. However, there still exists a research gap of investigating the mattering factors and driving mechanism promoting students’ learning persistence in online learning environments, which provides theoretical ground and practical reference for facilitating students’ learning persistence in the future. Transactional distance theory, developed from the work of Moore [4], is an applicable theoretical foundation to explain the antecedents and driving mechanism of learning outcomes, because students’ interaction has been proven to be a crucial predictor of learning persistence and academic achievement [5]. In addition, students’ interactions have a close relationship with emotional and social engagement [6] and sense of community [7], which is of great significance in effectively promoting the learning persistence [8]. According to the types of the interaction in online learning environments, the most eye-catching interaction is classified into three types, namely student-student (SS) interaction, student-instructor (SI) interaction, and student-content (SC) interaction [9,10,11]. These three types of interaction can provide emotional, instructional, and organizational support, further facilitating social engagement [6] and learning persistence [12]. Despite the significance of three types of interaction, very few empirical studies have explored the roles of three types of interaction for learning persistence in an online learning environment.

Prior studies stated that different types of academic emotions play crucial roles in students’ interaction and learning persistence [13]. For example, Huang et al. [14] revealed that positive academic emotions (e.g., enjoyment, happiness) might lead to more interaction between instructors and students. In contrast, negative academic emotions (e.g., anxiety, boredom) might hinder learning engagement [15]. As for learning persistence, Hwang [16] and Oh et al. [17] addressed that students’ interaction was associated with anxiety and learning persistence in online learning environments. Similarly, Baker et al. [18] found that a great variation in students’ academic emotions may significantly affect their learning persistence. Based on previous research results, academic emotions were identified as the main antecedence for students’ learning persistence. Besides, students’ academic emotions were employed as the moderators and mediators in the proposed research model to further explore the relationship between students’ interaction and learning persistence.

Few studies, however, have empirically explored the relationships among students’ interaction, academic emotions and learning persistence. The objective of this paper is to investigate the influence of students’ interaction on learning persistence from the perspective of moderation and mediation of academic emotions (enjoyment, anxiety and boredom) in online settings. To address these issues, three research questions are explored in this article: (1) How are students’ interaction and academic emotions (enjoyment, anxiety, and boredom) related to learning persistence in online learning? (2) Do academic emotions (enjoyment, anxiety, and boredom) mediate the relationship between students’ interaction and learning persistence in online learning? (3) Do academic emotions (enjoyment, anxiety, and boredom) moderate the relationship between students’ interaction and learning persistence in online learning?

## 2. Theoretical Background

The person-environment interaction model [19] and transactional distance theory [20] focus on the relationships among students’ interaction, academic emotions, and learning persistence. A brief review of them is described in this section.

### 2.1. Person-Environment Interaction Model

The interaction between an individual and an environment is considered as an extremely critical factor in understanding individuals and determining their behaviors [21]. The model of person-environment interaction proposed by Moos [22] focused on the bi-directional relationship between people and environments that is correlated to a range of various states in the psychological and physical domains, such as cognitive [23] and motivation [24]. In an online learning environment, based on person-environment interaction theory, Neufeld et al. [21] proposed that academic constructs (e.g., learning persistence and engagement) reflected the interaction between a student and his or her educational environment. Adopting the person-environment fit theory, Martin and Rimm-Kaufman [6] revealed that the lack of alignments between the needs of individual students and their learning environments results in the lower emotional and social engagement. In addition, by integrating the theoretical grounds of contemporary integral approaches and doctrine of reciprocal determinism, Dębek [25] extended an integrative transactional framework to elaborate the person-environment interaction including emotions, behaviors, and learning persistence. In line with the person-environment interaction theory, this study thereby theoretically proposed that students’ interaction and academic emotions could facilitate students’ learning persistence in online learning environments.

### 2.2. Transactional Distance Theory

Transactional distance theory is a well-established contemporary theory on the interplay between individuals, environments, and interaction patterns [26]. In online learning environments, this theory addresses the relationship between interaction patterns and pedagogical subjects [9]. It also specifically distinguishes three major aspects of interaction, namely SS interaction, SI interaction, and SC interaction. Those three types of interaction are seen as a necessary condition for offering worthwhile and meaningful online learning environments [27]. Gokool-Ramdoo [28] assumed that students’ interaction could lead to an effective educational transaction and facilitate learning persistence. He also highlighted that emotional factors (e.g., enjoyment, anxiety) should be considered. However, prior studies have been mainly confined to the macro dimension of emotional or interactional engagement. There is thus an urgent need to explore students’ learning persistence from the perspective of students’ multi-dimensional of interaction and academic emotions to improve students’ engagement and persistence. The provenances of the two theories mentioned above are different and complementary. They both affirm that students’ interaction, academic emotions and learning persistence are interrelated in online learning environments.

## 3. Literature Review

### 3.1. Learning Persistence

Learning persistence refers to students’ willingness to complete learning objects and tasks, such as obtaining degrees or completing courses content, which requires students to overcome obstacles that occur in the online learning process [2]. It has been recognized as a significant factor in successful learning. Related to learning persistence, Wong [29] articulated that students’ learning persistence or dropout was related highly to academic achievements. In the same vein, Fang et al. [30] used math learning log data to explore the relationship between patterns of persistence-related learning behaviors and academic performance during learning. Furthermore, Zhai et al. [31] proposed that students’ perceived satisfaction may significantly predict learning outcomes and behavioral intention of persisting with learning. It is therefore reasonable to assume that learning persistence is a valuable indicator for predicting learning outcomes and achievement. In addition to the relations between learning persistence and academic performance, we must also be aware of direct relations among factors such as students’ interaction and academic emotions in online learning environments. Oh and Lee [17] stated that three types of students’ interaction built a crucial relationship between learning-related anxiety and intention to persist among e-learning students with visual impairment. To conclude, previous findings suggested that learning persistence was a considerable factor in online learning environments.

### 3.2. Student Interaction in Online Learning

Students’ interaction is a key source of success in education context [6]. The conceptual framework of interaction developed by Moore posits three types of interaction, SS interaction, SI interaction, and SC interaction. According to the person-environment interaction model, students’ interaction has long been considered as one of the main pedagogical issues [32]. The interaction functions among student, instructor and content are complementary in online educational practice because interaction among students is supported by instructor facilitation and support, which in turn, centers on content [33]. Based on the person-environment interaction model and transactional distance theory, we, therefore, drew on the three types of interaction of Moore.

Numerous studies have shown a high prevalence of SS interaction in online learning environments [34]. SS interaction results from participation, communication and discussion between students in asynchronous or synchronous communication without the direct involvement of instructors. A study by Gray and DiLoreto [35] explained that students who had greater interaction with others in an online course achieved higher levels of perceived learning. Gašević et al. [36] proposed that graduate students could reach higher levels of knowledge construction and learning outcomes in student-student discussions. Borokhovski et al. [37] highlighted that the relationship between student-student interaction and collaborative learning in Interactive learning environments. Moreover, Kyei-Blankson et al. [38] concluded that student-student interaction was the most important form of interaction.

As SI interaction is a critical component of the online learning process [39], instructors provide motivational and emotional support in a way that can enhance and maintain the student’s interest. This is consistent with the fact that the most significant variable in an online course is students’ interaction with the instructor [40]. In addition, Dwyer [41] emphasized that student-instructor interaction could reap persistence and academic benefits. Moreover, Molinillo et al. [42] suggested that social presence and teacher-student interaction had a positive influence on students’ active learning, both directly and indirectly, through emotional engagement. A study by Cho and Jonassen [43] revealed that students who enjoyed interacting with others were more likely to have a high self-efficacy for interaction with their instructor. Overall, instructors can develop close interaction relationships and promote deeper levels of learning in online learning environments [44].

With the development of online learning, scholarly interest in SC interaction has also increased. Interaction with content refers to a one-way process of elaborating and reflecting on the subject matter or the course content. SC interaction may include reading informational texts, using study guides, discussing questions, and completing assignments. Through the interaction with content, students cognitively elaborate, organize, and reflect on the new knowledge by integrating previous knowledge [10]. Abulibdeh and Hassan [45] considered that SC interaction was the vital predictor of students’ academic achievement. Similarly, Ertmer et al. [46] agreed that one of the major success factors of online courses was effective SC interaction. In particular, Krudysz and McClellan [47] indicated that more focus on SC interaction could minus the loss in educational effectiveness. The ample aforementioned studies have discussed that all types of interaction promote learning engagement and persistence. Those findings provide a better perspective for exploring the relationships between students’ interaction and learning persistence.

### 3.3. Academic Emotions

Online learning is laden with intense emotional experience [48]. An increasing number of empirical studies have examined a range of academic emotions related to online learning. According to the control-value theory [49], academic emotions can be distinguished along dimensions of activation (activating vs. deactivating), valence (positive vs. negative), and object focus (activity vs. outcome). Although several different academic emotions can be induced in online learning conditions, the present study emphasizes the detailed effects of academic emotions (enjoyment, anxiety, and boredom) because they are the most frequently and intensely academic emotions experienced in online learning environments [13].

Enjoyment, anxiety, and boredom can be described as the major dimensions of academic emotions. As an activating positive activity-related emotion, enjoyment reinforces task activity, focuses attention on the task, and predicts students’ elaboration and metacognition positively [50], leading to the use of learning strategies [51] and even self-regulation [52]. Anxiety, defined as an activating negative outcomes-related emotion, was the most often reported emotion in many studies [53]. Academic anxiety arises when students believe their cognitive and/or motivational skills may be overwhelmed by the demands of a highly valued academic situation [54]. Numerous studies have demonstrated that anxiety was negatively correlated with learning performance [55] and self-efficacy [56]. Being treated as a deactivating unpleasant activity-related emotion, boredom undermines task incentives disrupts attentional focus [57] and leads to lower levels of motivation [58] and metacognition [59]. As stated in the literature, academic emotions (enjoyment, anxiety, and boredom) have different effects on academic performance and achievement.

Academic emotions are momentous factors in online learning environments. However, it is not probing the roles of different academic emotions in the relationship between students’ interaction and learning persistence.

### 3.4. Academic Emotions as Mediator and Moderator in Online Learning

Conforming to the social cognitive model, academic emotions act as mediators or moderator role in a learning process. You and Kang [60] examined the role of academic emotions (enjoyment, anxiety, and boredom) in the relationship between perceived academic control and self-regulated learning in online learning. The results supported that enjoyment had a mediating effect on the relationship between perceived academic control and self-regulated learning. Boredom and anxiety showed significant moderating effects on the relationship between perceived academic control and self-regulated learning and students who experienced low boredom/anxiety with high perceived academic control demonstrated high self-regulated learning. Similarly, Villavicencio and Bernardo [61] contended that enjoyment and pride both moderated the relationship between self-regulation and grades. For students who report higher levels of pride/enjoyment, self-regulation was positively related to grades. For students who had lower levels of pride, self-regulation was not related to grades. For those who reported lower levels of enjoyment, self-regulation was negatively associated with grades. Adapting transactional distance theory, Oh and Lee [17] described the buffering effect of three types of interaction in the relationship between learning-related anxiety and intention to persist with e-learning. The results indicated that students with higher levels of anxiety tended to have lower levels of intention to persist with e-learning.

Little research has addressed the mediating or moderating effect of academic emotions in the relationship between students’ interaction and learning persistence. As such, this study aims to examine the effect of academic emotion, by regarding it as a third variable in the relationship between students’ interaction and intention to persist with online learning.

### 3.5. Research Hypotheses

**Figure 1:**
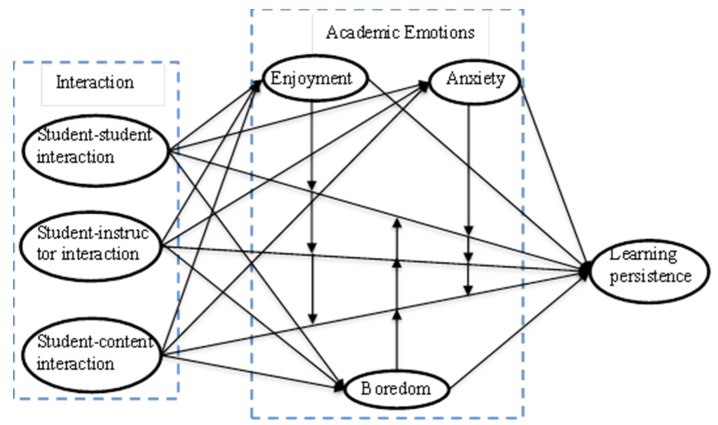
The proposed research model.

Based on the above-mentioned theories and literature, we aim to explore the relationship among students’ interaction, academic emotions and learning persistence in this study. As shown in Figure 1, three types of students’ interaction are hypothesized as playing a direct role in students’ academic emotions and their learning persistence. We hypothesized that three types of interaction play a positive role in predicting enjoyment and relate negatively to anxiety and boredom. We also hypothesized that three types of students’ interaction have positive relationships with their learning persistence. Also, based on theoretical background and literature review, three types of students’ interaction are assumed to predict learning persistence through the mediation or moderation of academic emotions. In addition, enjoyment is hypothesized as having positive relationships with learning persistence. On the contrary, anxiety and boredom are hypothesized as having negative relationships with learning persistence.

## 4. Method

### 4.1. Participants and Procedure

The study sample included 339 (response rate: 84%) participants who had online learning experience from five universities in China. Generally, the participants who had online learning experience need to watch the weekly scheduled online lecture videos and to complete the assignments. When a student posted a question related to the lectures, others and the instructors could start a discussion or reply to the question on the forum.

Participants were required to complete the questionnaire anonymously and voluntarily. All participants knew the research background and purposes, and provided their written informed consent before completing the measures in the study. In order to make sure everyone treated the survey seriously, the researchers offered a pen in return. In addition, this study has gained the approval from the ethical committee of five universities in China. The sample demographics are shown in Table 1.

### 4.2. Instruments

Three separate scales, namely the Online Interaction Scale (TOIS), the Online Emotional Scale (TOES), and the Online Learning Persistence Scale (OLPS), were utilized to measure students’ interaction, academic emotions, and learning persistence in the online learning environment. All the items in Chinese used a 5-point Likert scale ranging from 1 (completely disagree) to 5 (completely agree). Brief descriptions of the three scales are provided as follows.

#### 4.2.1. The Online Interaction Scale (TOIS)

A 19-item, three-factor scale used in this study was modified from that of Kuo [10] for investigating students’ interaction. The scale consisted of three distinct interaction subscales: student-student interaction with eight items (e.g., “I got lots of feedback from my classmates”), student-instructor interaction with six items (e.g., “I had numerous interactions with the instructor during the class”), and student-content interaction with five items (e.g., “Online course materials stimulated my interest for this course”).

#### 4.2.2. The Online Learning Persistence Scale (OESE)

The online learning persistence scale (OESE) developed by Shin [62] was adopted in this study to estimate students’ learning persistence in an online learning environment. The scale consisted of five items. A sample of such items was that “I will finish my online course no matter how difficult it may be.”

#### 4.2.3. The Online Emotion Scale (OLES)

The Online Emotion Scale (OLES) was adapted from the Achievement Emotions Questionnaire (AEQ) [63] for measuring academic emotions. Based on the literature review, we chose three emotional variables (enjoyment, anxiety, and boredom) that were relevant to online learning. Specifically, a four-item enjoyment subscale measured students’ enjoyment, with a sample item of “I enjoy my online course”. A four-item boredom subscale was used, such as “Because I am so bored during the online lecture, I frequently check the time”. A four-item was finally for assessing students’ anxiety. For example, “I was worried that I might say something wrong, so I’d rather not express my opinion.”

### 4.3. Data Analysis

In order to analyze the collected data, we used SPSS 22.0 (IBM, Armonk, NY, USA) and AMOS 22.0 (IBM, Armonk, NY, USA) [64]. First, the basic descriptive statistics of all the measured variables were conducted as a preliminary analysis. We then computed Cronbach’s alpha and used the data from the confirmatory factor analysis (CFA) to obtain the value of Composite Reliability (CR) and the Average Variance Extracted (AVE) [65]. Second, we utilized structural equation modelling (SEM) to test the mediation effects of enjoyment, anxiety, and boredom in the relationship between students’ interaction and learning persistence. This was achieved by following the two-step approach that estimated the measurement model and the path analysis results [66]. Using the hierarchical regression analysis in IBM SPSS, we finally performed a series of moderation analysis in order to validate the moderating effect of enjoyment, anxiety, and boredom.

According to the goodness-of-fit indices [67], chi-square (χ^2^), and chi-square divided by degrees of freedom (χ^2^/*df*), we verified the results by using the goodness of fit index (GFI), the comparative fit index (CFI), the standardized root mean square residual (SRMR) and the root mean square error of approximation (RMSEA). For both CFI and GFI, values with being greater than 0.90 indicate a good fit, while values below 0.08 for RMSEA and SRMR indicate an acceptable fit.

## 5. Results

### 5.1. Reliability and Validity of Measures

We calculated the descriptive statistics of each latent variables, the skewness and kurtosis coefficients ranging from −0.67 to 1.45, and the results supported the univariate normality assumption. In order to obtain good internal consistency and convergent effect, we conducted CFA to test the adequacy of latent variables. Due to different cross-cultural backgrounds, the items of three instruments were deleted based on the three criteria: (1) all of the item factor loadings should be higher than 0.6; (2) the values of the CR should exceed 0.8; (3) the values of AVE should exceed 0.5 [65], with each construct still having at least 3 items. As listed in Table 2, the Cronbach’s alpha (α) coefficients for all of these latent variables ranged from 0.78 to 0.96, the CR coefficients exceeded 0.70 (0.76–0.96), and Cronbach’s α and the CR of all the latent variables were higher than 0.70. All of these indicated that the internal consistency of all latent variables was high. In addition, the AVE exceeded 0.50 (0.50–0.85), implying that the measurement had a good convergent effect.

### 5.2. The Fitness of Model

First, we performed CFA to measure the fitness indices of the measurement model of each scale respectively. The CFA of students’ interaction revealed that the three-factor model fit the data well (χ^2^/*df* = 2.030, CFI = 0.961, GFI = 0.933, RMSEA = 0.055, and SRMR = 0.049). The fitness indices of the three-factor model for the academic emotions (χ^2^/*df* = 2.669, CFI = 0.977, GFI = 0.947, RMSEA = 0.070 and SRMR = 0.051) indicated a good model fit.

Then, the full measurement model included three latent constructs (students’ interaction, academic emotions and learning persistence), seven latent variables and 31 observed variables. The measurement model indices showed an excellent fit to the data: χ^2^/*df* = 2.429, CFI = 0.924, GFI = 0.958, RMSEA = 0.056 and SRMR = 0.050.

**Figure 2:**
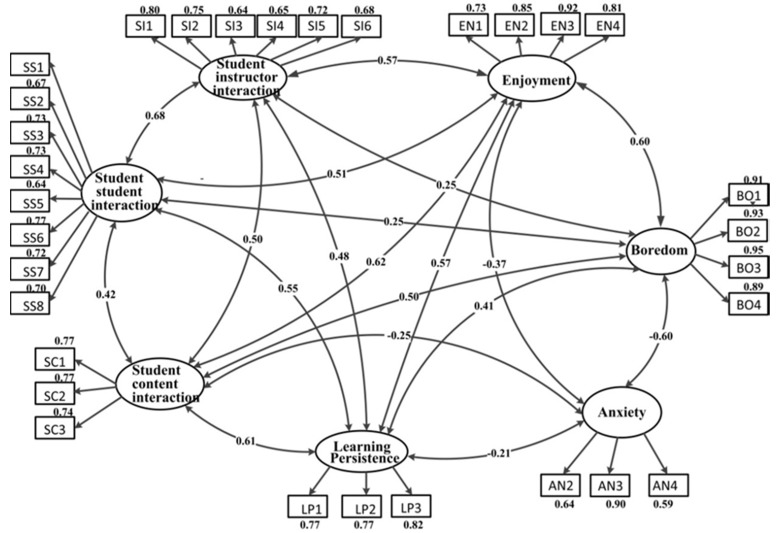
The measurement model (standardized factor loadings).

As shown in Figure 2, standardized factor loadings, which represented the relationships between each indicator and the corresponding latent variable, ranged from 0.59 to 0.95, indicating that all the latent constructs were well represented by their indicators. The correlations between latent variables were all significant (*p* < 0.001), except for the relationship between student-student interaction and anxiety (r = −0.058), and student-instructor interaction and anxiety (r = −0.033).

### 5.3. Mediating Effects of Emotions

In the section, we performed SEM to explore respectively the mediating effect of enjoyment, anxiety, and boredom on the relationship between students’ interaction and learning persistence.

#### 5.3.1. Mediating Effects of Enjoyment on Students’ Interaction and Learning Persistence

We corroborated the initial hypotheses on the relationships among students’ interaction, enjoyment and learning persistence. Both SS and SC interactions positively predicted enjoyment (*B* = 0.27, *p* < 0.001 for SS and *B* = 0.53, *p* < 0.001 for SC) and learning persistence (*B* = 0.31, *p* < 0.001 for SS, and *B* = 0.31, *p* < 0.001 for SC). So did SI interaction for enjoyment only (*B* = 0.26, *p* < 0.001). In addition, enjoyment predicted learning persistence (*B* = 0.22, *p* < 0.001) positively. Further, we tested the mediating effect of enjoyment by analyzing the indirect effect of students’ interaction on learning persistence. In particular, we measured the indirect effect using a bootstrapping method, with a bootstrapped 95% confidence interval. The results are summarized in Table 3. As shown in the table, both SS and SC interactions positively predict learning persistence both directly (*B* = 0.308, *p* < 0.001 for SS, and *B* = 0.314, *p* < 0.001 for SC) and indirectly (*B* = 0.057, *p* < 0.05 for SS, and *B* = 0.115, *p* < 0.01 for SC), mediated by enjoyment. These results indicate that partial mediation exists between SS interaction, SC interaction and learning persistence.

#### 5.3.2. Mediating Effects of Anxiety on Students’ Interaction and Learning Persistence

We verified the initial hypotheses on the relationships among students’ interaction, anxiety and learning persistence. Learning persistence was positively predicted by SS interaction (*B* = 0.36, *p* < 0.001), SI interaction (*B* = 0.17, *p* < 0.01), SC interaction (*B* = 0.42, *p* < 0.001). Particularly, anxiety negatively predict learning persistence (*B* = −0.18, *p* < 0.01). We also found that the three types of students’ interaction did not predict the mediating variable (anxiety) significantly. Therefore, we concluded that anxiety had no mediating effects between the relationship of students’ interaction and learning persistence in online learning environments. This is because there is an insignificant effect between the independent variable and mediator as suggested by Baron and Kenny [67].

#### 5.3.3. Mediating Effects of Boredom on Students’ Interaction and Learning Persistence

For testing the hypotheses on the relationships among students’ interaction, boredom and learning persistence, we have the following results. Learning persistence was positively predicted by SS interaction (*B* = 0.35, *p* < 0.001), SI interaction (*B* = 0.16, *p* < 0.01), and SC interaction (*B* = 0.36, *p* < 0.001). Specifically, boredom negatively predicted learning persistence (*B* = −0.17, *p* < 0.01), while SC interaction negatively predicts boredom. The mediating effect of boredom was further examined by analyzing the indirect effect of SC interaction on learning persistence. Table 3 summarizes the results. Mediated by boredom, SC interaction positively predicted learning persistence both directly (*B* = 0.357, *p* < 0.001) and indirectly (*B* = 0.075, *p* < 0.05). These results indicate that partial mediation exists between SC interaction and learning persistence.

### 5.4. Moderating Effects of Emotions

Using the hierarchical regression, we examined the respective moderating effect of enjoyment, anxiety, and boredom on the relationship between students’ interaction and learning persistence. As suggested by Aiken et al. [67], we first centralized the predictor and moderator variables by plus-minus one standard deviation (±1SD) of their respective mean. For testing the moderation effect, the variables were then entered into the model in the following three steps. Step 1 was the predictor three types of students’ interaction (SS interaction, SI interaction and SC interaction). Step 2 was the moderator of academic emotions (enjoyment or anxiety or boredom), followed by the interaction term in step 3. Hierarchical regression was conducted three times for three moderation models showed in Table 4. Models 1–3 captured the moderation effect for enjoyment on the relationship of students’ interaction and learning persistence. Models 4–6 for anxiety on the relationship of students’ interaction and learning persistence and Models 7–9 for boredom on the relationship of students’ interaction and learning persistence.

#### 5.4.1. Moderating Effects of Enjoyment on Students’ Interaction and Learning Persistence

In order to examine whether enjoyment moderated the relationship between students’ interaction and learning persistence, we used three types of interaction as the predictor variable, learning persistence as the dependent variable, and enjoyment as the moderator. As shown in Table 4, the results indicated that the predicted interaction terms between SI interaction and enjoyment were significant. This means that enjoyment significantly moderated the association between SI interaction and learning persistence. The result also showed that enjoyment was not a significant moderator for the relationship between SS interaction and learning persistence. Neither was for the relationship between SC interaction and enjoyment.

**Figure 3:**
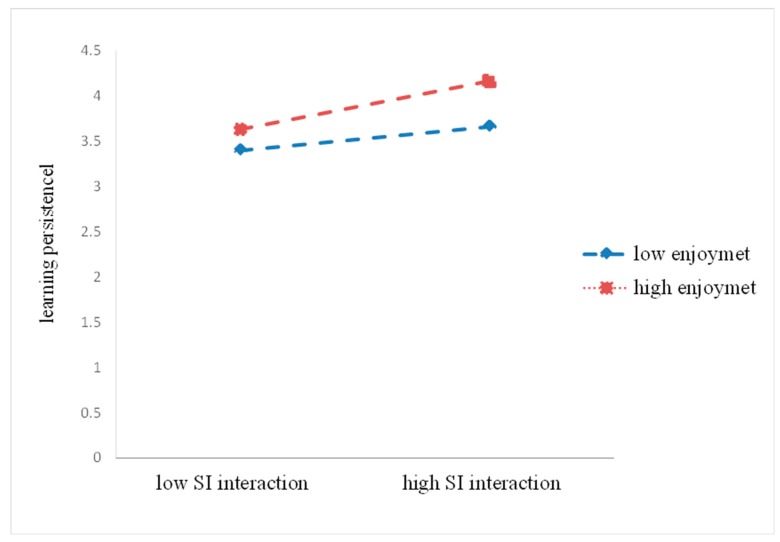
Enjoyment as a moderator of the relationship between SI interaction and learning persistence.

For elaborating on the moderating effect of enjoyment clearly, we obtained the simple regression slopes. It depicts the predicted relationship between the predictor variable and the dependent variable at the low and high levels of the moderator. With applying the plotting method recommended by Hayes et al. [68], ±1SD the mean was generally expressed at high and low levels. As shown in Figure 3, the slopes were different between the low and high enjoyment. This indicated that the association between SI interaction and learning persistence differed significantly at different levels of enjoyment. In particular, students who perceived high enjoyment and SI interaction showed high learning persistence

#### 5.4.2. Moderating Effects of Anxiety on Students’ Interaction and Learning Persistence

The moderation analysis examined whether anxiety moderated the relationship between students’ interactions and learning persistence. As reported in Table 4, the results indicated that the predicted interaction terms between SS interaction and anxiety, as well as between SI interaction and anxiety, were significant. It is interesting to note that anxiety was not a significant moderator of the relationship between SC interaction and learning persistence.

**Figure 4:**
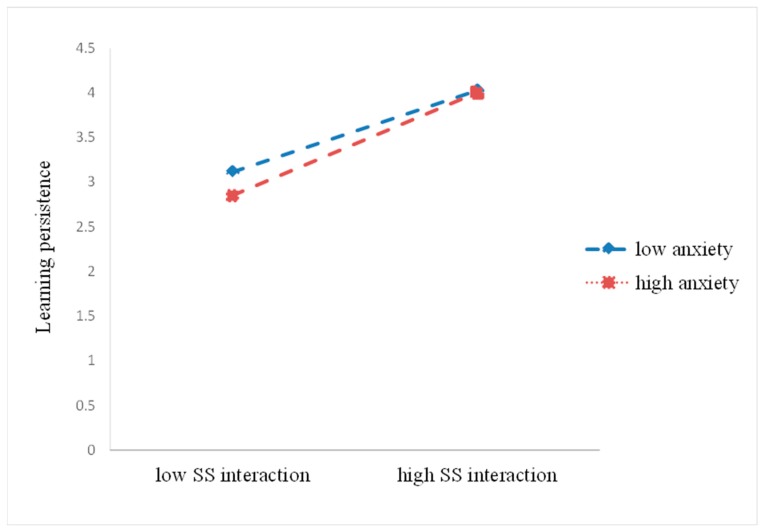
Anxiety as a moderator of the relationship between SS interaction and learning persistence.

**Figure 5:**
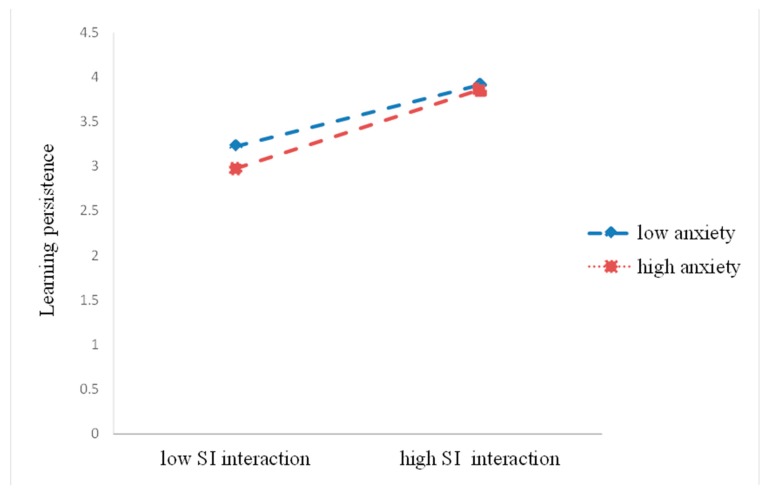
Anxiety as a moderator of the relationship between SI interaction and learning persistence.

In order to further examine the moderation effect of anxiety, we illustrated the simple regression slopes in Figure 4 and Figure 5 as before. The results demonstrated significant relationships between SS interaction and learning persistence at high and low levels of anxiety. In particular, the strength of the association between SS interaction and learning persistence increased as anxiety decreased. As for the relationship between SI interaction and learning persistence, the learning persistence of students who perceived a high level of anxiety was lower for those with lower SI interaction.

#### 5.4.3. Moderating Effects of Boredom on Students’ Interaction and Learning Persistence

To test the moderating effects of boredom between three types of interaction and learning persistence, we performed the same moderation analysis. As shown in Table 4, the results demonstrated that boredom significantly moderated the association between SS interaction and learning persistence. 

**Figure 6:**
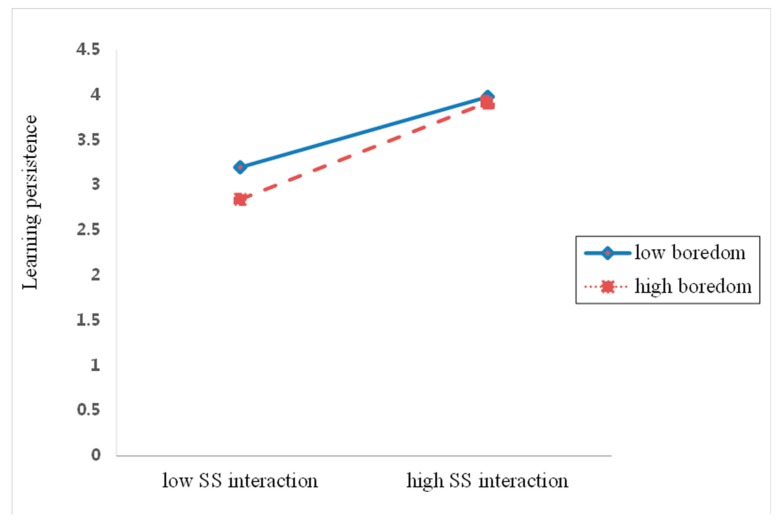
Boredom as a moderator of the relationship between SS interaction and learning persistence.

Once again, simple regression slopes were plotted in Figure 6 in order to further interpret the moderating effects of boredom. The slopes revealed that the association between SS interaction and learning persistence differed significantly in low and high levels of boredom. In particular, students with a high level of boredom and the low SS interaction experienced lower learning persistence. The result also suggested that boredom was not a significant moderator of either the relationship between SI interaction and learning persistence, or the relationship between SC interaction and learning persistence.

## 6. Discussion

In this study, we aimed to investigate the relationships among students’ interaction, academic emotions (enjoyment, anxiety, and boredom) and learning persistence in online learning environments. Our research elaborated three types of interaction and contributed to theoretically and empirically identifying different roles of academic emotions between students’ interaction and learning persistence. Overall, the study suggested that three types of interaction and academic emotions could effectively catalyze learning persistence and engagement in online learning environments. Those statistics results were consistent with previous research [15,41].

### 6.1. Relationships Among Students’ Interaction, Academic Emotions and Learning Persistence

For academic emotions, this study showed that all of the three types of interaction had a positive association with enjoyment experiences. The results corroborated and built on previous studies of students’ interaction in the online learning environment. According to the person-environment interaction model, for example, Caldwell [69] found that high levels of interaction between students and their environments (peers, instructors, and content) could produce an enjoyable learning atmosphere. We also found that only SC interaction negatively predicted boredom. This finding was consistent with that by Putwain et al. [13]. If learning materials are simple, students could be bored. In addition, students’ enjoyment positively predicted learning persistence. On the contrary, anxiety and boredom negatively predicted students’ learning persistence. These findings are consistent with those of Putwain [13] and Yang [15]. As stated before, students’ academic emotions should be considered to be critical in predicting their learning persistence.

### 6.2. Academic Emotions as Mediators

In line with our hypothesis, enjoyment mediated the relationships between SS interaction and learning persistence. The results were consistent with previous studies on highlighting the peers’ ability for the students’ learning persistence. Students were required to communicate and discuss with their peers in online learning environments to form positive attitudes [70]. Also, we found that enjoyment mediated the relationships between SC interaction and learning persistence. A possible explanation for this result might be that online learning environments may provide students with the platform to interact with the content of learning materials. As such, students cognitively elaborate, organize, and reflect on the new knowledge with their enjoyable experience. Finally, we found boredom could mediate the relationship between SC interaction and learning persistence. As a deactivating emotion, boredom leads learners to be disengaged [49]. Thus, Instructors need to assign interesting and challenging tasks to students.

### 6.3. Academic Emotions as Moderators

Another aim of this study was to estimate the moderation effect of academic emotions. Our moderation analysis indicated that the experience of enjoyment could moderate the relationship between SI interaction and learning persistence. The results further showed that students who perceived a high level of enjoyment, learning persistence were higher for those who reported a higher level of SI interaction. This is particularly important because enjoyment could strengthen the relationship between student and instructor so as to catalyze learning persistence or learning engagement [42].

In addition, our results demonstrated that anxiety moderated the relationship between SS interaction and learning persistence, as well as the association with SI interaction and learning persistence. Meanwhile, we also found that students who experienced low anxiety with high SS/SI interaction demonstrated high learning persistence. The finding supported Oh and Lee’s [17] study in the sense that students with higher levels of anxiety tended to have lower levels of their learning persistence. The high level of interaction with the instructor or students was not sufficient to facilitate learning persistence. This was perhaps because anxiety, an activating negative emotion, was induced if students may be struggling to build the relationships with instructors and peers as a result of the lack of immediate feedback in an online learning environment [71].

Another potentially important finding is that boredom moderated the relationship between SS interaction and learning persistence. The results also demonstrated that student who perceived a high level of boredom with low SS interaction demonstrated a low level of learning persistence. It is consistent also with the previous research concluded that a relationship between SS interaction and learning persistence varies greatly according to different levels of their boredom. Moreover, boredom has recently been identified as frequently experienced and extremely damaging emotion in an online learning environment [72]. Collectively, these findings validate the assumption that boredom (a negative deactivating emotion) may be associated more with the relationship between the SS interaction and learning persistence than anxiety (a negative activating emotion). This may be partial because negative deactivating emotions have stronger and potentially more harmful effects on learning persistence [64].

It is important to highlight that the moderating effect of academic emotions between SC interaction and learning persistence is slight. The lack of the significant moderating effect of academic emotions may be caused by several factors. According to the interaction equivalency theorem [73], in a case where no detrimental effects are on learning, and one of the types of interaction is at a high level, the other two types of interaction can be then at lower levels or even eliminated. In addition, it is not always easy to discern SC interaction. Other variables such as SS interaction or student-system interaction may be tied [74].

## 7. Practical Implications

The present findings have several practical implications. According to the person-environment interaction model and transactional distance theory, students’ interaction and academic emotions are two of the main sources of learning persistence.

First, academic emotions in this study have both mediation and moderation effects on the relationship between students’ interaction and learning persistence. The mediating and moderating effects may afford a possible interpretation for the non-significant relationship observed or mixed results [50]. This suggests that researchers should consider the joint relationship between academic emotions and students’ interaction when examining the relationship between students’ interaction and learning persistence.

Second, not only students’ interaction but also enhancing positive emotions and declining negative emotions are required to improve learning persistence. From mediation analysis, enjoyment mediated the relationships between SS interaction, SC interaction and learning persistence. Boredom mediated the relationships between SC interaction and learning persistence. From moderation analysis, the relationship between students’ interaction and learning persistence differ substantially with different levels of academic emotions. However, the finding showed that having a high level of students’ interaction was not sufficient to enhance learning persistence. The students who experienced high negative academic emotions (anxiety and boredom) and interacted with others at the high levels did not exhibit their high learning persistence. Therefore, reducing students’ negative emotions and enhancing positive emotions is of significance to promote their learning persistence [75].

Third, our results demonstrated that academic emotions (enjoyment, anxiety and boredom) produced the different mediating and moderating effect in the online learning process. Therefore, it is important to make diverse implications corresponding to a specific emotion. According to Pekrun’s [49] taxonomy of academic emotions, enjoyment is an activating positive activity-related emotion, which has a positive moderating relationship between SI interaction and learning persistence in the online learning process. In addition, enjoyment also mediate the relationships between SS interaction, SC interaction and learning persistence, Therefore, greater attention to enjoyment should be paid. Contrastingly, anxiety is regarded as an activating negative outcomes-related emotion, which leads to lower learning persistence for online students. In online learning environments, it is necessary to provide personalized, process-related assistance about learning material to reduce students’ anxiety. Boredom is treated as a deactivating unpleasant activity-related emotion, which leads students to be disengaged. Instructors need to provide interesting and useful learning tasks to their students so that communications among them can happen. Overall, increasing positive emotions and decreasing negative emotions are a fruitful way to buffer learning persistence and engagement.

Finally, the importance of providing interactional support to students should be emphasized. An instructor should focus more on students-oriented interactions by providing feedback on students’ work and informing their progress. In this way, the students are encouraged to actively participate in their course discussions [76]. Generally, the instructor should help students study in harmony and enjoy their learning activities so as to avoid or minimize negative emotions. As such, various modern communication technologies such as e-mails, online forums and social media can be employed to build a sense of an online community [77]. These technologies make it possible to create an atmosphere of the ease of interaction that allows students to persist with their learning. In a word, not only students’ academic emotions but also supportive interaction is malleable characteristics of learning persistence in online learning environments.

## 8. Conclusions and Limitations

This study provides a new perspective on understanding the mechanisms that underlie the relationship between students’ interaction, academic emotions and learning persistence. According to the person-environment interaction model and transactional distance theory, we attempt to establish the links among students’ interaction, academic emotions, and learning persistence. Our results have shown that academic emotions act as moderator and mediator between students’ interaction and learning persistence in online learning environments. To summarize, the present study offers empirical support for the relationship among students’ interaction, academic emotions and learning persistence. Meanwhile, the study further highlights the mediating and moderating effects of academic emotions when exploring the strength of relations between students’ interaction and learning persistence in an academic setting.

However, some of our findings should be interpreted with caution, as a result of the following limitations. First, the cross-sectional and correlational design used in this study does not consider a definitive determination of the causal relationships between variables. Although several empirical studies provide support for the proposed model, we cannot exclude the possibility of reciprocal relationships between variables Future research would design experiments and use qualitative methods for exploring the relationship among students’ interaction, academic emotions and learning persistence.

Second, the sample size of our research was relatively small. A larger number of samples could certainly increase the statistical power. Moreover, the generalization of our results could be limited as a result of our samples from universities, majors, and learning subjects only. Thus, our future studies should focus on hierarchical data to reduce the generalization error of the results.

Finally, our survey data relied on self-report instruments, which may result in the same-source bias. The results of this study could be further verified by using diverse methods such as interviews, open-ended questionnaires, and physiological data. The construct of emotion could be reported by biofeedback and physiological data, for example.

## Figures and Tables

**Table 1:** Sample demographics (*n* = 339).

Variables	Classification	Total (%)
Gender	Male	103 (30.4%)
	Female	236 (69.6%)
Age	Less than 18	7 (2.1%)
	18–25	282 (83.2%)
	26–30	41 (12.1%)
	More than 30	9 (2.6%)
Major	educational technology	145 (42.8%)
	computer science	113 (33.3%)
	communication	40 (11.8%)
	business	26 (7.5%)
	medicine	15 (4.6%)
Educational level	undergraduate	149 (44%)
	master	134 (39.5%)
	doctoral	11 (3.2%)
	other	45 (13.3%)

**Table 2:** Descriptive statistics and Cronbach’s α, CR, AVE of the variables (*n* = 339).

Variables	SS Interaction	SI Interaction	SC Interaction	Enjoyment	Anxiety	Boredom	Learning Persistence
**Mean**	3.32	3.25	3.50	3.36	2.74	3.60	3.58
**SD**	0.71	0.71	0.72	0.80	0.76	0.89	0.67
**Skewness**	−0.34	−0.29	−0.67	−0.45	0.30	−0.53	−0.25
**Kurtosis**	0.52	0.90	1.45	0.47	0.01	0.35	0.41
**α**	0.90	0.86	0.80	0.90	0.78	0.96	0.81
**CR**	0.89	0.86	0.80	0.90	0.76	0.96	0.83
**AVE**	0.50	0.50	0.58	0.68	0.52	0.85	0.62

**Table 3:** Direct, indirect effects on students’ interaction and learning persistence (*n* = 339).

Mediator	Predictor → Criterion	Direct Effect (p)	Indirect Effect	
			Sum (p) ^a^	CI ^b^
Enjoyment	Partial mediation			
	SS interaction → learning persistence	0.308 ***	0.057 *	0.006, 0.150
	SC interaction → learning persistence	0.314 ***	0.115 **	0.017, 0.241
Boredom	Partial mediation			
	SC interaction → learning persistence	0.357 ***	0.075 *	0.011, 0.168

Notes: (a) The probability associated with the sum of standardized indirect effects was estimated using the two-sided bias-corrected confidence interval bootstrap test of AMOS 22 (confidence level = 95%; samples = 5000). (b) CI = Confidence Interval. * *p* < 0.05; ** *p* < 0.01; *** *p* < 0.001.

**Table 4:** Moderating effort of academic emotions on students’ interaction and learning persistence.

Learning Persistence											
Variables	Model 1	Model 2	Model 3	Variables	Model 4	Model 5	Model 6	Variables	Model 7	Model 8	Mode l9
	Standardized Coefficient (Beta)				Standardized Coefficient (Beta)				Standardized Coefficient (Beta)		
**Independent Variables**				**Independent Variables**				**Independent Variables**			
SS	0.308 ***	0.265 ***	0.225 ***	SS	0.308 ***	0.301 ***	0.264 ***	SS	0.308 ***	0.295 ***	0.272 ***
SI	0.081	0.022	0.020	SI	0.081	0.083	0.087	SI	0.081	0.079	0.112
SC	0.326 ***	0.238 ***	0.255 ***	SC	0.326 ***	0.320 ***	0.304 ***	SC	0.326 ***	0.269 ***	0.247 ***
**Moderator**				**Moderator**				**Moderator**			
Enjoyment (EN)		0.226 ***	0.234 ***	Anxiety (AN)		−0.136 ***	−0.145 ***	Boredom (BO)		−0.153 ***	−0.170 ***
**Interaction Variable**				**Interaction Variable**				**Interaction Variable**			
SS × EN			−0.037	SS × AN			−0.164 ***	SS × BO			−0.204 ***
SI × EN			0.172 ***	SI × AN			−0.178 ***	SI × BO			−0.100
SC × EN			−0.016	SC × AN			−0.018	SC × BO			−0.051
R	0.578	0.602	0.617	R	0.578	0.589	0.613	R	0.578	0.595	0.611
R^2^	0.334	0.362	0.381	R^2^	0.334	0.347	0.376	R^2^	0.334	0.354	0.373
ΔR^2^	0.334	0.028	0.019	ΔR^2^	0.334	0.013	0.029	ΔR^2^	0.334	0.019	0.020
F	56.093 ***	47.418 ***	29.135 ***	F	56.093 ***	47.225 **	41.189 **	F	56.093 ***	45.692 ***	28.167 ***

Note: *N* = 339. SS: student-student interaction; SI: student-instructor interaction; SC: student-content interaction; EN: enjoyment; AN: anxiety; BO: boredom. ** *p* < 0.01; *** *p* < 0.001.
